# Concerns on a new therapy for severe heart failure using cell sheets with skeletal muscle or myocardial cells from iPS cells in Japan

**DOI:** 10.1038/s41536-018-0047-2

**Published:** 2018-03-21

**Authors:** Yoshiki Yui

**Affiliations:** Kyoto, Japan

## Abstract

In Japan, a new therapy for severe heart failure using skeletal myocardial sheet is ongoing and a treatment using iPS cells is just about to start. However, I think there are problems and concerns. In this comment, I will express concerns on these treatments. This will be useful and interesting to stem cell researchers and clinical cardiologists.

Induced pluripotent stem (iPS) cells are a promising source of cells for regenerative medicine, and we have high expectations. Biomedical engineering with cell sheets is an interesting approach for the treatment of severe heart failure.

In this comment, I will express my concerns about an ongoing and upcoming new therapy in Japan for severe heart failure using cell sheets with skeletal myoblasts or iPS cells.

## Autologous skeletal myoblast sheets

On 1 October of last year (2017) at the 65th Annual Scientific Session of the Japanese College of Cardiology (Osaka), Prof. Y. Sawa (Department of Cardiovascular Surgery, Osaka University) gave a special lecture. The topic was on autologous skeletal myoblast sheets for the treatment of severe chronic heart failure. He also presented a new therapy with induced pluripotent stem (iPS) cells instead of skeletal myoblasts as the next stage of treatment.

I have the following concerns regarding this therapy based on his published article,^1^ the product document https://www.pmda.go.jp/safety/info-services/ctp/0001.html created by Terumo Corporation authorized by PMDA (Pharmaceuticals and Medical Devices Agency, Japan), a council report,^2^ and his lecture.

1. In the study, the left ventricular ejection fraction (LVEF)^3^ was employed as the primary endpoint.
  The change in LVEF was classified as improved (ΔLVEF ≥ 5%), unchanged (5% > ΔLVEF > −3%), or worsened (−3% ≥ ΔLVEF). The corresponding number of patients were 0, 5, and 2, each respectively.
  In Fig. 2 of the paper,^1^ the LVEF between pretreatment and 26 weeks after the surgery is shown, but the statistics were not described. Using the data in the product document https://www.pmda.go.jp/safety/info-services/ctp/0001.html, I performed a statistical calculation with the paired *t*-test. The two-tailed *P*-value was 0.6985 (not statistically significant). I have two serious questions about this study. First, the LVEF as a primary endpoint is unreliable and inappropriate, because heart failure with preserved ejection fraction exists.^4^ Second, among the 7 patients, 5 were classified as “responders” to this therapy. However, the definition of “responders” to this therapy appears medically inappropriate, because “responders” included those with unchanged LVEF (5 patients) in addition to patients with improved LVEF (0 patients). As pointed out in the council report (Page 58),^2^ the fluctuation (−5–5%) in LVEF is a measurement error. This council report recommends that ΔLVEF ≥ 5% should be the improvement of cardiac function.
2. In the product document, the indication for using autologous skeletal myoblast sheets is as follows:

- Patients with severe heart failure due to ischaemic heart disease.
- Patients who did not respond to the standard therapies (drugs and interventional devices).
- NYHA (New York Heart Association) class III or IV^5^
- LVEF at rest is 35% or less.

In the above lecture, Prof. Y. Sawa presented 12 cases after 6 months of the operation, and he concluded that, for patients with LVEF less than 30%, this sheet seems to have little effect in improving severe heart failure. Considering the above inclusion criteria (2)-d, the number of patients with the indication seems to be very small.

Currently, the clinical trial with skeletal myoblast sheets is ongoing. In September, 2015 using the above 7 cases, the Terumo Corporation obtained a temporary approval from the Ministry of Health, Labour and Welfare. However, within 5 years, after completing additional 60 cases with ischaemic heart failure, an application for the final approval must be submitted. The superiority of the survival rate compared with 120 cases with standard therapies is required. This requirement by PMDA seems highly plausible. However, this trial is not a placebo-controlled, double-blind RCT (randomized-control trial). Patients backgrounds are different. Even if propensity score matching is used, evaluating the effectiveness of the trial may be difficult.

In Japan, regenerative medicine is controlled by Pharmaceuticals and Medical Devices Act, the Act to Ensure the Safety of Regenerative Medicine, and the Clinical Trial Act. The Clinical Trial Act was legislated last year (2017) by the Diovan (Valsartan) misconduct, which I pointed out.^6,7^ Under Pharmaceuticals and Medical Devices Act, a fast track conditional approval (conditional time-limited authorization) started. This autologous skeletal myoblast sheets was the first approval.

## iPS cell sheets

On July 19 of last year (2017), there was an official announcement from the Graduate School of Medicine, Faculty of Medicine, Osaka University about a new project by Prof. Y. Sawa involving the treatment of severe heart failure with iPS cells http://www.med.osaka-u.ac.jp/archives/7911. In the statement, it was mentioned that the autologous skeletal myoblast sheets was not effective for more severe heart failure. Hence the use of myocardial cells from iPS cells is being developed alternatively. In the above meeting, Dr. Sawa explained the protocol using iPS cells instead of skeletal myoblasts. However, these iPS cardiomyocytes were not derived from the patient’s own cells but were from stocked cells from other donors. Stocked iPS cells from healthy donors with homozygous human leucocyte antigen, for which safety is checked beforehand are going to be used.^8^ This modified strategy with allotransplantation is due to the cost and time issues associated with autologous iPS cells.^8^ This type of iPS cells requires immunosuppressive drugs. The expectation had been raised that autologous cells would be used for the cell sheets. This is apparently not the case and this should be made entirely clear by the team involved. The use of autologous iPS cells is hopefully a future perspective that will be realized.

According to Dr. Sawa and the above statement, he will start this new surgical therapy as early as this year to evaluate the safety of iPS-derived myocardial cells. Unlike other iPS trials, such as those for Parkinson’s disease and age-related macular degeneration of the eye, the objective evaluation of improvement of severe heart failure is far more difficult. To obtain a statistically adequate number of patients and assess them during the follow-up period, an international multicentere trial supported by the Japanese government is absolutely necessary. This trial with iPS cardiomyocyte sheets is interesting and challenging. If properly developed, this iPS therapy would surely open a new era in the treatment of severe heart failure.

Hopefully, before beginning the new therapy with iPS cell sheets, extensive and precise data about ongoing trials with skeletal myoblast sheets will be released.

In conclusion, for trials dealing with severe heart failure, strict statistics, protocols and hard endpoint (death) are necessary:

1. The primary endpoint should be cardiac and total death.
2. A placebo-controlled double-blind RCT is ideal.

During a pilot study phase, placebo and double-blind are not necessary. However, at final stage, to prove the effectiveness of the treatment, both are necessary, which will finally lead to the interests of patients.

If hard endpoint (death) and surrogate endpoints^9^ including subjective symptoms are dramatically improved, a placebo-controlled double-blind RCT may become unnecessary.
