# Author Correction: Concerns on a new therapy for severe heart failure using cell sheets with skeletal muscle or myocardial cells from iPS cells in Japan

**DOI:** 10.1038/s41536-018-0051-6

**Published:** 2018-07-06

**Authors:** Yoshiki Yui

**Affiliations:** Kyoto, Japan

**Correction to**: *npj Regenerative Medicine* 10.1038/s41536-018-0047-2, Published online 21 March 2018

The Author Affiliation was incorrect in the original paper.

Yoshiki Yui is unaffiliated (Kyoto, Japan).

This error has now been corrected in the HTML and PDF.

